# Establishment of a differential diagnosis method and an online prediction platform for AOSD and sepsis based on gradient boosting decision trees algorithm

**DOI:** 10.1186/s13075-023-03207-3

**Published:** 2023-11-16

**Authors:** Dongmei Zhou, Jingzhi Xie, Jiarui Wang, Juan Zong, Quanquan Fang, Fei Luo, Ting Zhang, Hua Ma, Lina Cao, Hanqiu Yin, Songlou Yin, Shuyan Li

**Affiliations:** 1 The First Clinical College of Xuzhou Medical University, Xuzhou, 221004 China; 2 Department of Rheumatology and Immunology, Affiliated Hospital of Xuzhou Medical University, Jiangsu Province, China; 3 School of Medical Information and Engineering, Xuzhou Medical University, Xuzhou, 221004 China

**Keywords:** AOSD, Sepsis, Discriminant model, Machine learning, Gradient boosting decision tree

## Abstract

**Objective:**

The differential diagnosis between adult-onset Still’s disease (AOSD) and sepsis has always been a challenge. In this study, a machine learning model for differential diagnosis of AOSD and sepsis was developed and an online platform was developed to facilitate the clinical application of the model.

**Methods:**

All data were collected from 42 AOSD patients and 50 sepsis patients admitted to Affiliated Hospital of Xuzhou Medical University from December 2018 to December 2021. In addition, 5 AOSD patients and 10 sepsis patients diagnosed in our hospital after March 2022 were collected for external validation. All models were built using the scikit-learn library (version 1.0.2) in Python (version 3.9.7), and feature selection was performed using the SHAP (Shapley Additive exPlanation) package developed in Python.

**Results:**

The results showed that the gradient boosting decision tree(GBDT) optimization model based on arthralgia, ferritin × lymphocyte count, white blood cell count, ferritin × platelet count, and α1-acid glycoprotein/creatine kinase could well identify AOSD and sepsis. The training set interaction test (AUC: 0.9916, ACC: 0.9457, Sens: 0.9556, Spec: 0.9578) and the external validation also achieved satisfactory results (AUC: 0.9800, ACC: 0.9333, Sens: 0.8000, Spec: 1.000). We named this discrimination method AIADSS (AI-assisted discrimination of Still’s disease and Sepsis) and created an online service platform for practical operation, the website is http://cppdd.cn/STILL1/.

**Conclusion:**

We created a method for the identification of AOSD and sepsis based on machine learning. This method can provide a reference for clinicians to formulate the next diagnosis and treatment plan.

## Key message

The differential diagnosis between AOSD and sepsis has always been a challenge.

Delayed treatment of AOSD may lead to serious complications.

There are no studies on machine learning to distinguish AOSD from sepsis

## Background

Adult-onset Still’s disease (AOSD) is a systemic, inflammatory, and immune disease with unknown etiology and pathogenesis, of which clinical features are arthritis/arthralgia, high fever, transient rash, sore throat, and elevated ferritin [1, 2]. Because of the lack of specific symptoms and indicators, the diagnosis of AOSD remains a challenge. In clinical practice, patients with AOSD presenting with systemic inflammatory response syndrome as the first symptom are often difficult to distinguish from infectious diseases, especially septicemia [3, 4]. In recent years, many scientists have focused on the differential diagnosis of AOSD and sepsis, hoping to find satisfactory biomarkers to distinguish them. Some common hematological markers have been reported, such as neutrophil index, platelet/platelet distribution width, platelet/mean platelet volume, and red blood cell distribution width, can be used as single or supplementary indicators for the differential diagnosis of AOSD and sepsis [3, 5–7], However, the value of the single indicator for the differential diagnosis of AOSD and sepsis is limited, and the diagnostic performance can be improved when combined with other indicators. Delayed diagnosis of AOSD may lead to delayed treatment and serious complications, which may even be life-threatening. Therefore, rapid and accurate differential diagnosis of AOSD and sepsis is an urgent clinical problem to be solved.

Although AOSD is a relatively rare multi-system disease, with the continuous improvement of people's understanding of AOSD, the incidence and prevalence of AOSD are also gradually increasing [8]. At the same time, it has been recognized that various complications of AOSD include myocarditis, cardiopulmonary shock, multiple organ failure, joint deformity, macrophage activation syndrome (MAS), and acute respiratory distress syndrome. Macrophage activation syndrome (MAS) is one of the most serious and potentially life-threatening complications of AOSD, with a reported mortality rate of 20–40% [8–10]. Inadequate early inflammation control is associated with severe complications of AOSD [9]. Therefore, the early treatment of AOSD should attract our attention. Before this, differential diagnosis of AOSD is important.

Hematological indicators can quickly and easily reflect the situation of various diseases. Current studies have shown that many hematological indicators may be used as differential diagnostic markers for AOSD and sepsis, which also provides a basis for the development of diagnosis and differential model research based on laboratory indicators. With the development of medical and health data, the general statistical methods have been difficult to meet our needs for data analysis. At this time, machine learning methods have been more widely used in medicine. Many scholars have applied machine learning to the diagnosis or differential diagnosis of heart disease [11], diabetes [12, 13], COVID-19 [14], thyroid nodules [15], gastric cancer [16], and other diseases and achieved satisfactory results. However, there is no study on the use of machine learning methods to distinguish AOSD from sepsis.

In conclusion, there is a need to develop a novel and rational method to differentiate AOSD from sepsis. This study will compare three machine learning methods, including random forest (RF), gradient boosting decision tree (GBDT), and linear regression (LR), to combine the clinical features and hematological indicators and construct a differential diagnosis model for AOSD and sepsis. Finally, GBDT can select the best model, and the best model can be put on the website http://cppdd.cn/STILL1/, which is convenient for early and rapid identification of AOSD and sepsis in clinical practice.

## Methods

### Source of materials

All data were collected from 42 patients with AOSD and 50 patients with sepsis admitted to the Affiliated Hospital of Xuzhou Medical University from December 2018 to December 2021, of which 80% were used as the training set, and the other 20% were used as the test set. A total of 81 indicators including basic clinical characteristics, blood routine, liver function, renal function, immune series, coagulation function, erythrocyte sedimentation rate, and ferritin were collected. Pearson correlation coefficient method was used to eliminate indicators with high correlation (*R* > 0.90) and missing values ≥ 30%, and 70 indicators were finally left. The missing values were filled by the median or mean of the sample. In addition, 5 patients with AOSD and 10 patients with sepsis diagnosed in our hospital after March 2022 were collected for external validation. All AOSD patients were newly diagnosed and treated.

Inclusion criteria for AOSD patients are as follows: (1) age ≥ 18 years old; (2) meeting Yamaguchi’s diagnostic criteria [17]; (3) patients were initially diagnosed with AOSD and treated for the first time. Exclusion criteria are as follows: (1) co-infection, cancer, receiving chemotherapy, glucocorticoids, and other confounding factors of autoimmune diseases or treatments that may affect hematological parameters. Inclusion criteria for sepsis are as follows: (1) age ≥ 18 years old; (2) fulfilled the Third International Consensus Definitions for Sepsis and Septic Shock (Sepsis-3) [18]. Exclusion criteria are as follows: (1) critically ill patients; (2) confounding factors of cancer, chemotherapy, glucocorticoids, and other autoimmune diseases or treatments that may affect hematological parameters. Yamaguchi’s diagnostic criteria [17]: meeting at least five criteria and having two or more major criteria. The main criteria included (1) fever > 39 ℃ for at least one week; (2) arthralgia or arthritis lasting at least 2 weeks; (3) typical rash; and (4) white blood cell count ≥ 10 × 10^9^/L and granulocyte at least 80%. Secondary criteria included (1) sore throat; (2) splenomegaly/lymphadenopathy; (3) lack of RF or antinuclear antibodies; and (4) impaired liver function.

Written informed consent for all data was obtained from patients during their hospitalization, and the Ethics Committee of the Affiliated Hospital of Xuzhou Medical University approved the study.

### Key feature selection method

#### Introduction to the SHAP method

SHAP (Shapley Additive exPlanation) is a “model explanation” package developed in Python for feature selection that works on the principle of building an additive explanation model. For each predicted sample, the model produces a predicted value, and the SHAP value is the assigned value of each feature in the sample, reflecting the influence of each feature in the sample, and showing the positive and negative influence.

#### Introduction to the GBDT method

**Fig. 1:**
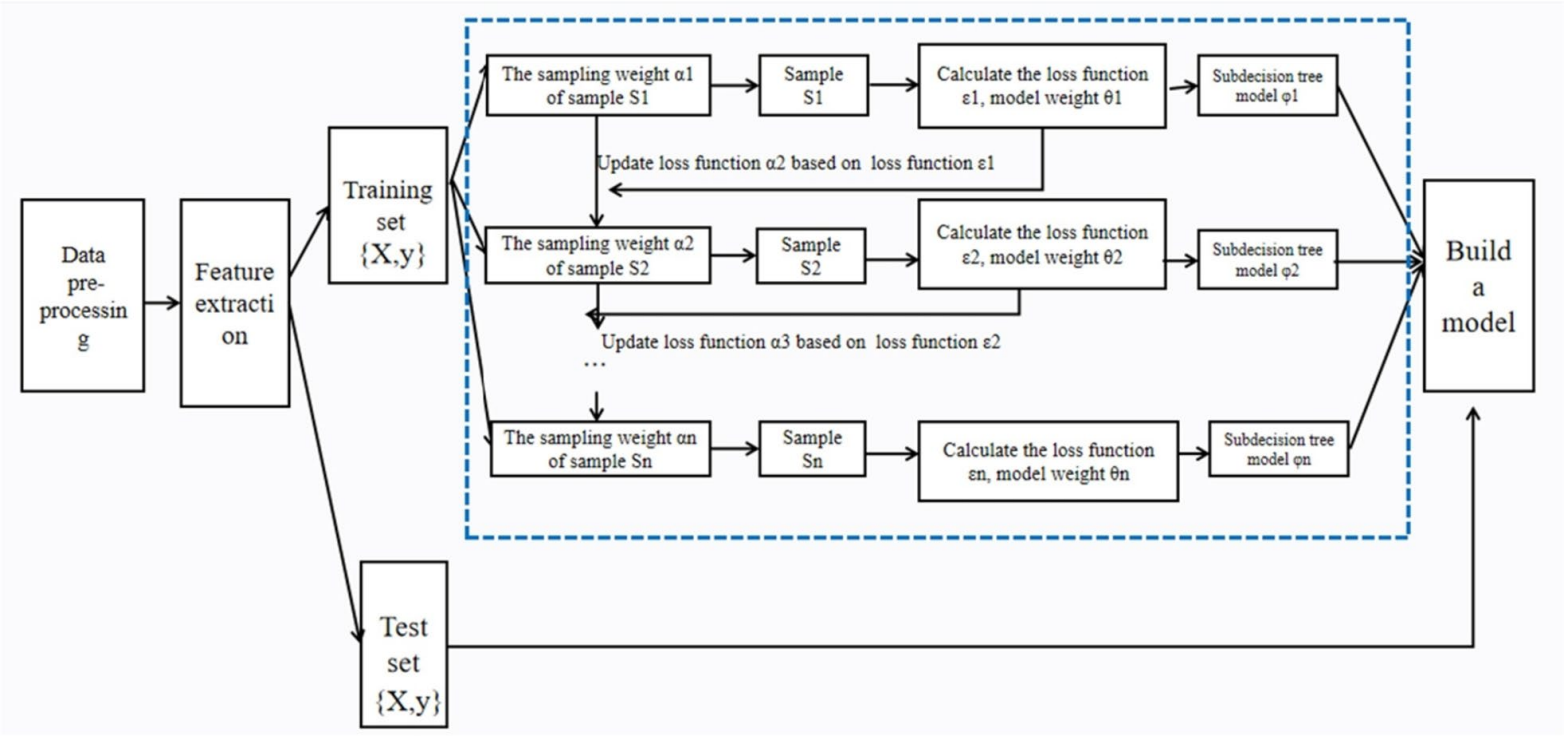
Schematic diagram of the gradient boosting tree model. Figure 1 illustrates the process of GBDT generating the model

Gradient Boosting Decision Trees (GBDT) is an ensemble learning algorithm that, as it runs, generates a final model based on a series of individual models, usually decision trees [19]. GBDT constructs the model using only a series of small decision trees at a time, each containing several variables from the total pool of variables studied [20]. These decision trees are built in an iterative fashion, splitting the data into smaller groups using cutoffs and then splitting the resulting groups again using another decision or cutoffs, so that the model built with the initial decision tree has residuals on which GBDT fits subsequent decision trees [20]. The prediction performance of each decision tree was relatively weak. However, when all decision trees are combined into the final model, the prediction performance is greatly improved [20]. The principle flow diagram of the gradient boosting decision tree operation is shown in Fig. 1.

All models were built using the scikit-learn library (version 1.0.2) in Python(version 3.9.7), and feature selection was performed using the SHAP (Shapley Additive exPlanation) package developed in Python.

### Model training and feature selection

**Fig. 2:**
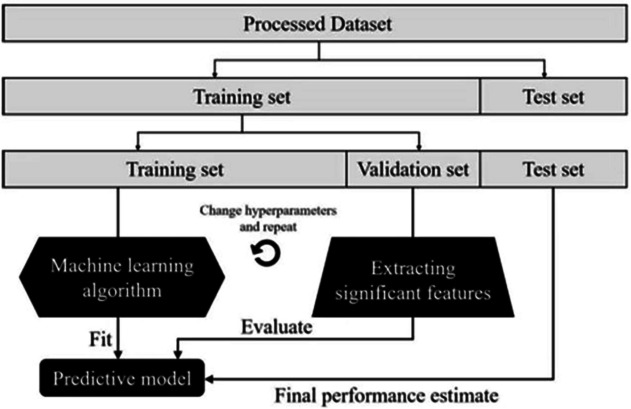
Model screening flowchart. Figure 2 shows the process of training and validating the model using machine learning

Model training and the final confirmation of the feature selection model are divided into three stages. The flow chart of machine learning is shown in Fig. 2. Firstly, the data were divided into a training set and test set at a ratio of 8:2. Logistic regression, random forest, and gradient boosting decision tree were used to train the data, respectively. On the basis of fivefold cross-validation, the prediction model was established by using a gradient boosting decision tree (GBDT) algorithm, and the gradient boosting decision tree was established as the final prediction model, and the importance of each feature related to the prediction result was demonstrated to select the most appropriate number of features and form the best prediction performance. In the second stage, on the basis of the original data set, we sequentially performed the product and ratio of each index to generate a total of 4230 new indicators. We used the same method as in the first stage to establish the prediction model and extract features, and also extracted 6 features. In the final stage, we combined the 12 features extracted from the above two stages to form a new dataset, re-established the model for feature screening, and screened out the final 5 features.

**Fig. 3:**
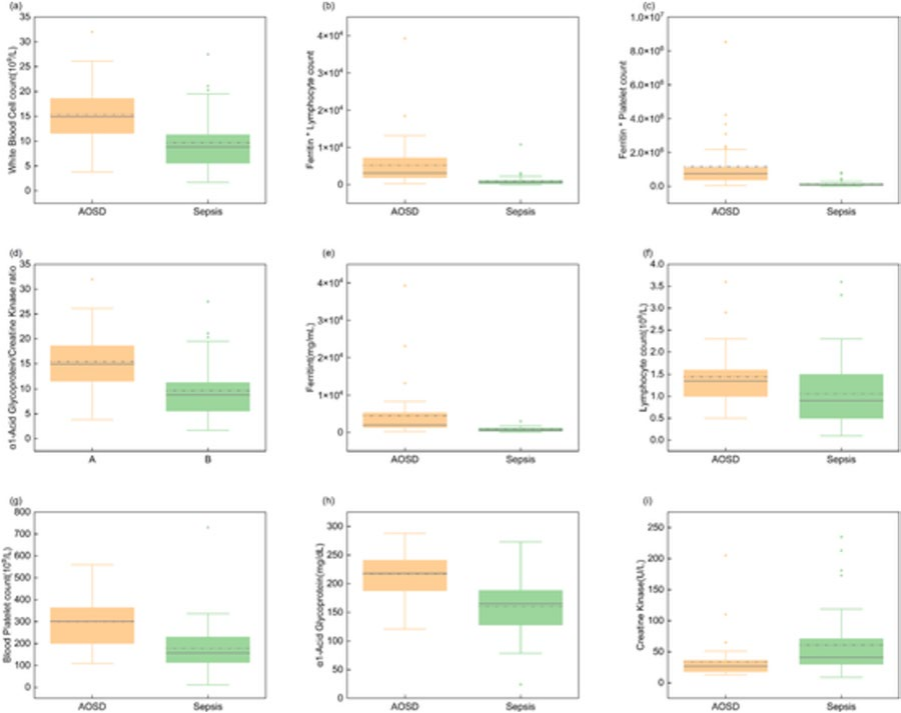
Significantly descriptors among AOSD and Sepsis. The figure shows the comparison of several important indicators of the differential model between AOSD and sepsis. It can be seen from the figure that the white blood cell count, ferritin * lymphocyte count, ferritin * platelet count, α1-acid glycoprotein/creatine kinase, ferritin, lymphocyte count, platelet count, α1-acid glycoprotein of AOSD are higher than those of sepsis. But creatine kinase in sepsis was higher than that in AOSD

In the second stage, various models were constructed using the adjusted parameters, which were based on increasing the number of top-ranked features. The hyperparameters of the model were adjusted by using the grid search method and the results of fivefold cross-validation. During this process, the number of n_estimators is adjusted from 93 to 81. Once the value of n_estimators is fixed, max_depth is updated from 9 to 4. In the final stage, according to the principle of the lowest number of top-ranked features and the same prediction performance compared with all features, the final model consists of five top-ranked features, including arthralgia, ferritin × lymphocyte count, white blood cell count, ferritin × platelet count, and α1-acid glycoprotein/creatine kinase. The distribution box diagram is shown in Fig. 3, where 0 represents sepsis and 1 represents Still’s disease.

### Methods of validation

In this study, the validation set and external validation were used to evaluate the model. In order to ensure the accuracy of the algorithm and the reliability of the model, and considering the small sample size, a fivefold cross-validation method was used for model validation. The data set was randomly divided into five non-overlapping parts, four of which were used as the training set and one as the validation set. Repeating this process for five times was called fivefold cross-validation, so that each sample could be used as a validation set. Finally, the performance of the model was re-evaluated using an external validation set.

It is also necessary to comprehensively evaluate the model using commonly used metrics such as sensitivity (SENS), specificity (Spec), accuracy (ACC), Matthews correlation coefficient (MCC), and AUC. These metrics can be calculated using true positive (TP), true negative (TN), false positive (FP), and false negative (FN) [16]:

$$\begin{array}{c}Sens=TP/(TP+FN)\\ \mathrm{Spec}=\mathrm{TN}/(\mathrm{TN}+\mathrm{FP})\\ \mathrm{ACC}=(\mathrm{TP}+\mathrm{TN})/(\mathrm{TP}+\mathrm{TN}+\mathrm{FP}+\mathrm{FN})\\ \mathrm{MCC}=(\mathrm{TP}\times \mathrm{TN}-\mathrm{FP}\times \mathrm{FN})/\sqrt{(\mathrm{TP}+\mathrm{FP})(\mathrm{TP}+\mathrm{FN})(\mathrm{TN}+\mathrm{FP})(\mathrm{TN}+\mathrm{FN})}\end{array}$$

The receiver operating characteristic(ROC) curve is often used to evaluate the diagnostic value of the model, and in general, a larger area under the ROC curve (AUC) indicates a higher diagnostic value of the model and a better prediction performance [16].

## Results

### Feature selection

**Fig. 4:**
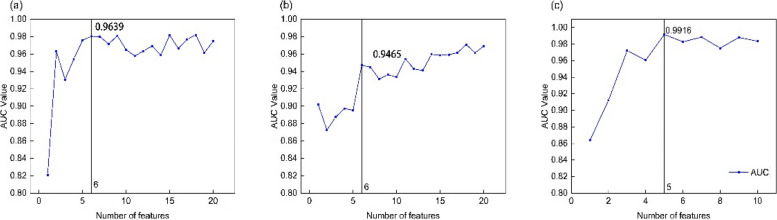
Comparison of AUC values of important features screened in three stages. **a** In the first phase, The SHAP method was used for important feature screening based on RF. white blood cell count, arthralgia, monocyte percentage, α1-acid glycoprotein, ferritin, and sore throat. The AUC value of the model was 0.9639. **b** In the second stage, the SHAP method, also based on RF, was used to screen out 6 important features: ferritin × platelet count, ferritin × lymphocyte count, ferritin × total protein, ferritin/urea, ferritin × erythrocyte sedimentation rate, and α1-acid glycoprotein/creatine kinase. The AUC value of the model was 0.9456. **c** In the third stage, on the basis of GBDT, the SHAP method was used to screen out the final 5 important features: arthralgia, ferritin × lymphocyte count, white blood cell count, ferritin × platelet count, α1-acid glycoprotein/creatine kinase. The AUC value of the model was 0.9916

We used the SHAP method to screen for important features. In the initial stage, white blood cell count, arthralgia, monocyte percentage, α1-acid glycoprotein, ferritin, and sore throat were selected. As shown in Table 1 and Fig. 4a. The prediction model established by GBDT had the best effect (AUC: 0.9755, ACC: 0.9324, Sens: 0.9600, Spec: 0.9017). In the second stage, after all the indicators were processed by ratio products, ferritin × platelet count, ferritin × lymphocyte count, ferritin × total protein, ferritin/urea, ferritin × erythrocyte sedimentation rate, and α1-acid glycoprotein/creatine kinase were selected. As shown in Table 2 and Fig. 4b. The AUC: 0.9573, ACC: 0.8781, Sens: 0.9270, Spec: 0.8678, by comparison, it was found that the prediction efficiency of the model was not as good as that of the first stage. Finally, the 12 indicators selected in the above two stages were further screened, and finally, our prediction model was selected, with only 5 indicators: arthralgia, ferritin × lymphocyte count, white blood cell count, ferritin × platelet count, α1-acid glycoprotein/creatine kinase, (AUC: 0.9916, ACC: 0.9457, Sens: 0.9556, Spec: 0.9578). The feature selection of the third stage and the feature selection AUC of the three stages are shown in Table 3 and Fig. 4c.

**Table 1:** Selected top 6 features in the first stage

Order	Features	Shap-Importance
1	White blood cell count	0.048411
2	Arthralgia	0.039684
3	Monocyte percentage	0.035879
4	α1 acid glycoprotein	0.028332
5	Ferritin	0.025614
6	Sore throat	0.024988
7	Creatinine	0.022643
8	Lactate dehydrogenase	0.020723
9	Percentage of eosinophils	0.019778
10	APTT	0.017245

*APTT* activated partial thromboplastin time

**Table 2:** Selected top 6 features in the second stage

Order	Features	Shap-Importance
1	Ferritin × platelet count	0.011470
2	Ferritin × lymphocyte count	0.008339
3	Ferritin × total protein	0.008214
4	Ferritin/urea	0.007739
5	Ferritin × ESR	0.007728
6	α1-acid glycoprotein/creatine kinase	0.007360
7	Lymphocyte count/MCV	0.005866
8	α1 acid glycoprotein/PDW	0.005416
9	Creatinine/urea	0.005194
10	α1-acid glycoprotein/age	0.005180

*ESR* erythrocyte sedimentation rate, *MCV* mean corpuscular volume, *PDW* platelet distribution width

**Table 3:** Selected top 5 features based on GBDT in the third stage

Order	Features	Shap-Importance
1	Arthralgia	1.5255
2	Ferritin × lymphocyte count	1.2290
3	White blood cell count	1.1873
4	Ferritin × platelet count	1.0806
5	α1-acid glycoprotein/creatine kinase	0.7984
6	Ferritin × ESR	0.7849
7	Ferritin/urea	0.7571
8	Sore throat	0.5257
9	Ferritin	0.3128
10	Monocyte percentage	0.2719

*ESR* erythrocyte sedimentation rate

### Model comparison and validation

**Fig. 5:**
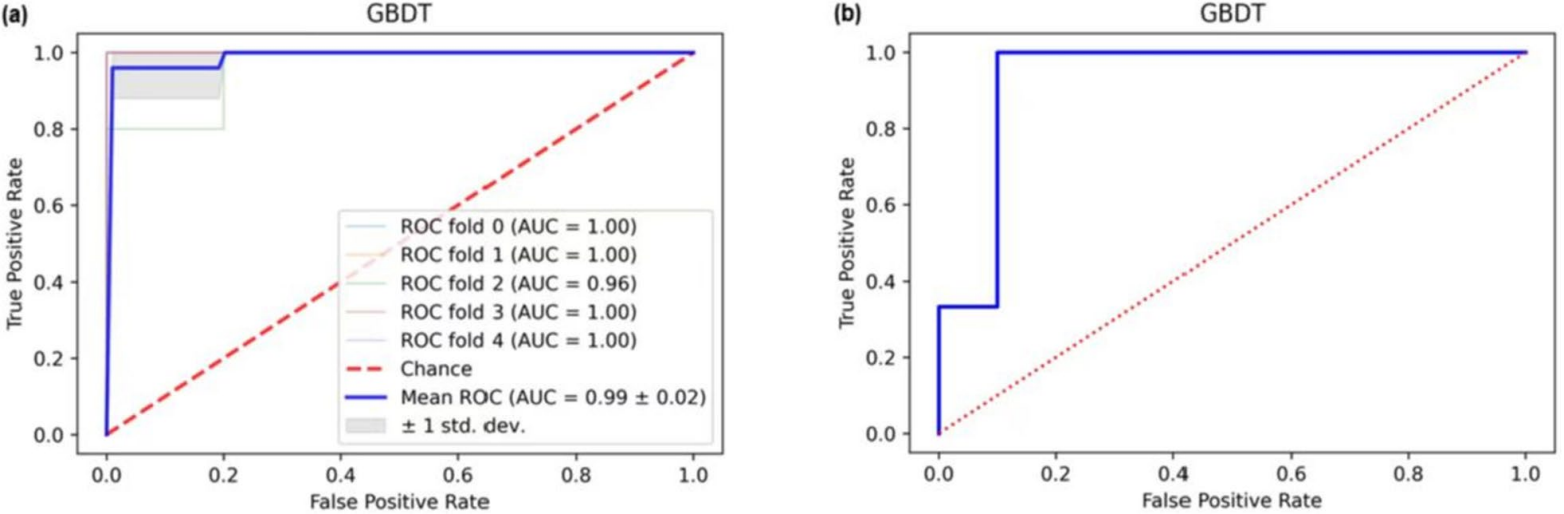
**a** ROC curve of GBDT model with fivefold interaction test. **b** ROC curve of the external validation set of the GBDT model

The prediction models generated in each stage were compared using three machine learning methods: RF, GBDT, and LR. Table 4 presents a comparison of the models generated in the third stage using the three machine learning methods. The prediction model formed by the GBDT method in the third stage had the best performance (AUC: 0.9916, ACC: 0.9457, Sens: 0.9556, Spec: 0.9578) and the least indicators. We also performed external validation, and finally achieved good prediction performance (AUC: 0.9800, ACC: 0.9333, Sens: 0.8000, Spec: 1.000). The ROC curves of the GBDT prediction model and external validation are shown in Fig. 5.

**Table 4:** Model comparison of three machine learning methods in the third phase

	**RF**		**GBDT**		**LR**	
	Test set	Validation set	Test set	Validation set	Test set	Validation set
AUC	0.9222	0.9832	0.9222	0.9916	0.8333	0.9229
ACC	0.8947	0.9171	0.8947	0.9457	0.7895	0.8229
Sens	0.8889	0.9667	0.8889	0.9556	0.7778	0.8814
Spec	0.9000	0.8737	0.9000	0.9578	0.8000	0.8050
MCC	0.7889	0.8422	0.7889	0.8981	0.5778	0.6864

Abbreviations: *AUC* area under curve, *ACC* accuracy, *Sens* sensitivity, *Spec* specificity, *MCC* Matthews correlation coefficient

### Prediction tool development

**Fig. 6:**
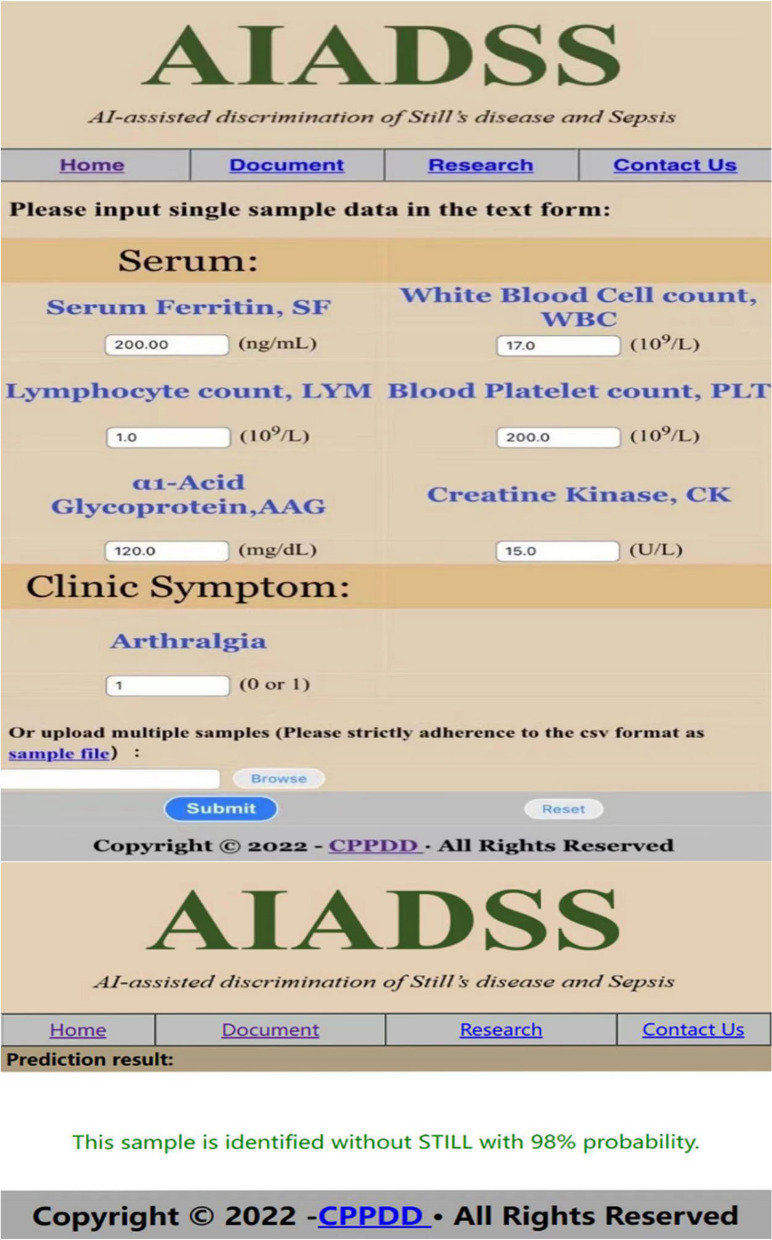
Schematic diagram of the AOSD versus sepsis discrimination model web page calculator. Users only need to input the relevant indicators of the subjects on the above page and submit them, and they can quickly make a preliminary differential diagnosis of AOSD and sepsis

Finally, in order to facilitate clinical use and promotion, we used the five indicators of arthralgia, ferritin × lymphocyte count, white blood cell count, ferritin × platelet count, and α1-acid glycoprotein/creatine kinase to establish a differential diagnosis model of AOSD and sepsis, and named it as AIADSS (AI-assisted discrimination of Still’s disease and sepsis). At the same time, we designed a website called http://cppdd.cn/STILL1/ on the web page, as shown in Fig. 6. The website is simple and fast. Users only need to enter the indicators of the model into the designated location on the website in order, and then click “Submit.” In the process of input, you should be careful that the input units are consistent with the units in the interface. After calculation and analysis, our model will conclude on the results page that the sample is AOSD or sepsis with a percentage probability.

## Discussion

Adult-onset Still’s disease (AOSD) is a systemic, autoinflammatory disorder that was first described in the early 1970s [21]. Most patients with AOSD present with high fever, transient rash, arthralgia or arthritis, and sore throat [21, 22]. The clinical features of AOSD are extremely similar to those of sepsis, also known as “Subacute septicemia,” especially in the early stage when fever is the initial clinical manifestation, it is often difficult to make a differential diagnosis between the two. Severe complications of AOSD are often associated with poor early inflammation control. However, there is no specific method to distinguish AOSD from sepsis at an early stage, resulting in delayed diagnosis and treatment [8, 23]. Currently, although some biomarkers have been explored for differentiating between AOSD and sepsis, none of them possess specificity. So far, there is no reliable model for discriminating between AOSD and sepsis, and there is also no reported application of machine learning methods in establishing a discrimination model. Therefore, we established a differential diagnosis model by combining common clinical features and laboratory tests and screened features by comparing three machine learning methods, including RF, GBDT, and LR.

The establishment of the model is a process of gradual exploration. In the initial stage, white blood cell count, arthralgia, monocyte percentage, α1-acid glycoprotein, ferritin, and sore throat were selected. The prediction model established by GBDT had the best effect (AUC: 0.9755, ACC: 0.9324, Sens: 0.9600, Spec: 0.9017). This model has preliminarily achieved satisfactory results in the differential diagnosis of AOSD and sepsis and has high sensitivity and specificity. In order to use fewer indicators to obtain better prediction effects, we also selected 6 features after the product ratio processing of all indicators in the second stage. The AUC: 0.9573, ACC: 0.8781, Sens: 0.9270, Spec: 0.8678, by comparison, it was found that the prediction efficiency of the model was not as good as that of the first stage. Therefore, we continued to explore, combine, and further screen the features extracted in the first two stages, and finally established a model with fewer indicators (5 indicators: arthralgia, ferritin × lymphocyte count, white blood cell count, ferritin × platelet count, α1-acid glycoprotein/creatine kinase) and higher prediction efficiency (AUC: 0.9916, ACC: 0.9457, Sens: 0.9556, Spec: 0.9578).

Arthralgia is one of the common symptoms of AOSD, and the commonly affected joints are the knee, wrist, ankle, elbow, and proximal interphalangeal joints [8, 10, 24–26], which is characterized by mild symptoms in the early stage and easy to be ignored. Sepsis often causes arthralgia because of joint or muscle infection and is characterized by typical joint symptoms of redness, swelling, heat, and pain, although the incidence of arthralgia in sepsis is small [27]. Arthralgia symptoms of AOSD can be relieved with the decrease of body temperature, but arthralgia in sepsis has no such characteristics. White blood cell count, a commonly used predictor of inflammation, is increased in both AOSD and sepsis. So far, there is no report that white blood cell count can be used to distinguish AOSD from sepsis, but white blood cell count was selected in our model. In the report by Fautrel B. et al., ferritin and glycosylated ferritin can be used for the diagnosis of AOSD, and glycosylated ferritin ≤ 20% can be used as one of the diagnostic criteria for AOSD [28, 29]. However, ferritin is increased in diseases such as infectious diseases and tumors, and when ferritin is used alone as a diagnostic marker, the specificity for the diagnosis of AOSD is poor, regardless of the threshold used [30]. Glycosylated ferritin is not readily available in most Settings and is therefore not practical in clinical practice. Zhang M et al. found through a retrospective study that lymphocyte count may be used as one of the indicators for the differential diagnosis of AOSD and sepsis, but the AUC of lymphocyte count alone was only 0.6760, and when it was combined with thrombocytocrit and ferritin, the AUC was 0.8360, the specificity was 0.9230, but the sensitivity was only 0.6730 [4]. Ge S. et al. suggested that platelet count to thrombocytocrit ratio (PMR) could be used as one of the differential diagnosis indicators of AOSD and sepsis. However, in the validation set, the AUC, sensitivity, and specificity of PMR alone as a differential diagnosis indicator were only 0.712, 0.8889, and 0.4286, even if PMR and ferritin were combined, all the evaluation indexes were improved, but the effect of differential diagnosis between AOSD and sepsis was still not satisfactory [6]. In our model, the related indexes discussed above also appeared, but they appeared in the form of products or ratios, such as ferritin × lymphocyte count, ferritin × platelet count, etc. Either alone or in combination, the performance of the above indicators in differentiating AOSD from sepsis was lower than that of the model established by GBDT (AUC: 0.9916, ACC: 0.9457, Sens: 0.9556, Spec: 0.9578).

First identified in 1950, α1-acid glycoprotein is produced mainly by the liver and some extrahepatic tissues and is increased in disease states such as infection, inflammation, and cancer [31–33]. α1-acid glycoprotein is a commonly used diagnostic biomarker [34]. Connelly M. A. et al. suggested that α1-acid glycoprotein can be used as a useful indicator to assess the activity of some autoimmune diseases [35]. Sun Y. et al. found that urinary α1-acid glycoprotein levels were significantly higher in AOSD patients than in non-AOSD patients [36]. In a prospective study, Ipek IO et al. found that two consecutive α1-acid glycoprotein measurements had a high sensitivity in the early diagnosis of neonatal sepsis, but a single α1-acid glycoprotein measurement had limited diagnostic value [37]. All these evidences indicate that α1-acid glycoprotein plays an important role in the diagnosis of inflammatory and autoimmune diseases, but there is no report on α1-acid glycoprotein used in the differential diagnosis of AOSD and sepsis.α1-acid glycoprotein was included in our first model screening. In our second model screening, although the single index of α1-acid glycoprotein was removed, the α1-acid glycoprotein/creatine kinase feature appeared. Therefore, we suggest that α1-acid glycoprotein plays an important role in the differential diagnosis of AOSD and sepsis but further studies are needed to confirm this.

Creatine kinase (CK) is found primarily in cardiac muscle, skeletal muscle, and brain tissue, with smaller amounts also found in lung, gastrointestinal tract, and thyroid tissues, which release sufficient amounts to increase their activity when diseased [38]. Creatine kinase elevation may occur in disease states such as muscle injury, brain tissue injury or tumor, hypothyroidism, and toxic effects of statins. Current studies indicate that myocardial dysfunction and prolonged muscle weakness are major causes of critical illness and death from sepsis [39–41]. Elevations in creatine kinase are often observed in patients with sepsis during cardiac injury and during skeletal muscle ischemia caused by sepsis-related hypotension [42–44]. Although some AOSD patients may present with muscle pain, there is no evidence that AOSD can cause muscle and myocardial damage, and the correlation between AOSD and creatine kinase has not been reported. This index was not screened in the first single index model, but appeared in the form of α1-acid glycoprotein/creatine kinase in the second multiple index product ratio. Therefore, whether creatine kinase can be used alone as a differential diagnosis between AOSD and sepsis is uncertain.

The limitations of this study include the following: first, it is a retrospective study, and all patients’ medical history data were obtained from the internal electronic medical record system of our hospital, which may not be accurate in the collection and recording of medical history, so information bias cannot be avoided. Secondly, due to the limitation of clinical sample size in our hospital, although the conclusions obtained by the statistical methods used in this study have good accuracy, the reliability needs to be further investigated. We plan to continue to expand the sample size in the future, and hope to further verify the results in clinical practice to make the conclusions more accurate. Finally, the differential diagnosis between AOSD and sepsis has always been a difficult problem to be solved. There may be other influencing factors that have not been further explored in the study, and further exploration is needed in the future.

## Conclusions

In summary, we used gradient boosting decision tree (GBDT) to screen features and establish a model for the differential diagnosis of AOSD and sepsis. The model consisted of arthralgia, ferritin × lymphocyte count, white blood cell count, ferritin × platelet count, and α1-acid glycoprotein/creatine kinase. Some of these indicators have been well-known or studied, such as ferritin, lymphocyte count, platelet count, etc. At the same time, some new potential indicators have also emerged, such as α1-acid glycoprotein and creatine kinase. Our team believes that this model is accurate, rapid, and simple to distinguish AOSD from sepsis, which can provide a reference for clinicians to make further diagnoses and treatment plans.

## Data Availability

All the data in this study came from the Affiliated Hospital of Xuzhou Medical University, the dataset can be accessed at https://doi.org/10.6084/m9.figshare.22117556
